# Police violence reduces trust in the police among Black residents

**DOI:** 10.1371/journal.pone.0308487

**Published:** 2024-09-11

**Authors:** Jonathan Ben-Menachem, Gerard Torrats-Espinosa

**Affiliations:** Department of Sociology, Columbia University, New York, New York, United States of America; University of Alabama in Huntsville, UNITED STATES OF AMERICA

## Abstract

Recent high-profile incidents involving the shooting or killing of unarmed Black men have intensified the debate about how police violence affects trust in the criminal justice system, particularly among communities of color. In this article, we propose a quasi-experimental design that leverages the timing of the shooting of Jacob Blake by the Kenosha Police Department relative to when a large survey was fielded in the city of Chicago. We demonstrate that individuals interviewed 4 weeks before and 4 weeks after the shooting are comparable across a large set of observed characteristics, thus approximating an experimental setting. We find that Blake’s shooting caused substantial reductions in Black respondents’ trust in the police, concentrated among younger residents and criminalized residents. These results suggest that police violence against racial minorities may lead to lower civic engagement and cooperation with law enforcement in those communities, exacerbating issues of public safety and community well-being. The pronounced distrust among younger Black residents suggests a generational rift that could risk further entrenching systemic biases and inequalities within the criminal justice system. Additionally, the higher levels of distrust among criminalized respondents could have implications for research detailing this population’s decreased willingness to engage with public institutions more broadly.

## Introduction

In recent years, the escalation of high-profile incidents of police violence, particularly against members of the Black community, has brought police violence to the forefront of domestic policy debates. From 2015 to 2020, there were 4,740 civilians fatally shot by police in the U.S., with a disproportionately high 26.7% being Black, while Hispanic fatalities stood at 18.8%, and white fatalities accounted for 51% of these cases [1]. This over-representation of minorities in fatal police encounters signifies a distressing trend that has made police violence a leading cause of death among young minority men [2].

The publicized killings of individuals such as Eric Garner, Philando Castile, George Floyd, and Jacob Blake have not only captured national attention but have also sparked a reevaluation of the role of law enforcement in society. Such incidents, often documented on video and spread across social media and news outlets, provide a raw glimpse into the interactions between police officers and Black individuals, potentially influencing public perceptions and trust in the police as an institution. These highly publicized events of police violence can change police-community dynamics. Individuals who have not been victims of police violence themselves may lose trust in law enforcement if they perceive the police as a discriminatory institution that systematically targets their racial group. This cycle of mistrust can result in a downward spiral where a heightened sense of alienation fuels more violence, particularly in the communities where police violence is high. Prior research finds that individuals reporting low trust in police are more likely to acquire a firearm for self-protection, either from nearby residents or from the police themselves [3, 4].

The relationship between law enforcement and the communities they serve has been a focal point of sociological research for decades [5–7]. Central to this discourse is the issue of trust in the criminal justice system, particularly among communities of color. Legal cynicism, or the belief in the incompetence and illegitimacy of the criminal justice system, is prevalent in many minority communities [8]. This cynicism, exacerbated by incidents of police violence, can lead to a reluctance to cooperate with law enforcement agencies.

Prior research has examined the extent to which police violence and misconduct perpetuate legal cynicism using interviews and correlational survey designs, finding that police contact can alienate residents who are subjected to it [9–11]. A growing number of studies have relied on 911 calls to document how city- and neighborhood-level patterns of crime reporting decline following police violence [7, 12]. Despite their important contributions, efforts to identify causal effects of police violence events have limitations. 911 calls may not be a reliable proxy to capture underlying trust in law enforcement. Furthermore, such studies often rely on data at the neighborhood or area level. The use of aggregate data poses challenges for causal inference and prevents the assessment of heterogeneous effects across different demographic groups.

The present study investigates how highly publicized events of police violence change trust in law enforcement, examining effect heterogeneity across race and ethnicity, age, and contact with the criminal legal system. These features of our research design could have implications for behaviors beyond cooperation with law enforcement such as system avoidance [13] or political participation [14].

To estimate the causal effect of police violence on trust in law enforcement, we leverage individual-level survey data in a quasi-experimental design that exploits the timing of the shooting of Jacob Blake (on August 23, 2020) relative to the fielding of the 2020 Healthy Chicago Survey (HCS), a survey of Chicago residents that the Chicago Department of Public Health ran between June 27, 2020 and December 9, 2020. Due to sample size limitations that prevent us to test effects by age, gender, and prior arrest record for other racial groups, our study primarily focuses on Black and White individuals. That being said, we do show results of an analysis of all Hispanic respondents in the S1 File. We show that individuals interviewed 4 weeks before and 4 weeks after the shooting took place are statistically identical. This enables a research design in which the group surveyed 4 weeks before the events serves as a plausible counterfactual for the group surveyed 4 weeks after.

We find large declines in trust in law enforcement among Black residents in Chicago after the shooting of Jacob Blake. During the two weeks that followed the shooting, trust in law enforcement declined by an average of 15 percentage points, a 31-percent decline from its baseline before the shooting. For Hispanic and white residents, trust in law enforcement remained unchanged. Among Black young adults (ages 18–44), law enforcement trust declined by up to 32 percentage points, with this effect lasting for three weeks. Among Black individuals who reported prior contact with the criminal legal system (e.g., having been arrested, booked, or charged at some point in the past), law enforcement trust declined by up to 36 percentage points in the two weeks following the shooting.

Our focus on individuals with prior exposure to policing and incarceration has theoretical and methodological importance; data limitations have made it difficult for researchers to identify the causal effect of police violence on institutional trust for this group. Generating causal evidence on the impact of police violence on trust is particularly important given the growing evidence showing that aggressive policing practices and police violence disproportionately affect people of color, low-income individuals, and other marginalized groups [15–17]. Accordingly, for sociologists of punishment, our findings constitute new evidence regarding the influence of police violence on perceptions of police among the most-policed communities. For political sociologists and political scientists, we contribute to a fast-growing literature showing how the behavior of “street-level bureaucrats” (rather than e.g. Presidential candidates) can shape citizens’ orientation toward government institutions [14].

## Literature and theory

American trust in police has been historically theorized via police legitimacy, or a person’s linked perception of law enforcement and their own obligation to obey the law [11]. Legitimacy theory is complemented by the concept of legal cynicism, a cultural frame in criminalized communities holding that law enforcement agencies are illegitimate, inadequate, or otherwise harmful [8, 18]. Bell further develops the concept of legal estrangement, wherein the experience of living in heavily policed communities reinforces a broader sense of marginalization at the hands of the law [19].

Empirical work measuring these interrelated concepts historically involved interviews or surveys digging into the mechanisms that drive e.g. Black Americans’ comparatively lower trust in police [5, 20]. Many such studies examine the connection between legitimacy and gun violence [3, 21]. A related body of work incorporates administrative data to assess whether events that negatively affect perceptions of police can also reduce residents’ willingness to report crimes or cooperate with law enforcement. This measure is consistent with the logic of police legitimacy, i.e., people who distrust the police are less likely to perceive reporting a crime to police as a viable solution. Desmond and coauthors found that calls for service in Milwaukee were reduced following the police beating of Frank Jude [7]. While this finding has been revised [22, 23], recent research found a similar negative effect following the murder of George Floyd [12].

This literature overlaps with work on social organization and collective efficacy, or a group’s ability to achieve collectively desired goals [24, 25]. Work in this spatial-ecological tradition has examined the connection between differential rates of interpersonal violence and interrelated measures of police legitimacy and willingness to enact informal social controls. Police violence may hinder collective efficacy in part by eroding trust in law enforcement and government institutions, particularly in communities where police violence is more prevalent.

Two recent studies provide strong evidence regarding the causal effects of police violence on trust. White and coauthors leverage a quasi-experimental design to assess the effect of the police killing of Freddie Gray on Baltimore residents’ perceptions of police legitimacy, but did not observe changes in perceptions of police. The authors suggest that this could follow from the lack of video footage depicting police brutality [26]. Additionally, their sample was not intended to be representative of Baltimore residents, but instead clustered in “hot spots.” Reny and Newman use a regression discontinuity design with nationally representative survey data to gauge the effect of George Floyd protests on perceptions of police, finding that protests diminished police favorability reports among politically liberal and “low-prejudice” respondents [27]. Although this study focuses on the effects of police brutality protests rather than police violence itself, these phenomena are tightly linked.

Apart from studies examining trust in police, recent work has improved the scholarly understanding of behaviors which follow from criminal legal contact (including police violence). Scholars argue that routine encounters with law enforcement constitute significant learning experiences with respect to government and one’s relationship with it [14, 28]. Political science studies detailing these dynamics generally contribute to prior theories of political socialization and focus on outcomes including voting or other political behaviors like volunteering for political candidates. Such work finds that many forms of criminal legal contact reduce voter turnout, often with pronounced effects for Black or Latinx residents [29–31]. This work focuses on personal and proximal contact–being personally arrested or learning about the arrest of a friend or family member. Yet criminal legal contact may also affect perceptions of police through less direct channels. To that end, Morris and Shoub theorize “community contact”: “diffuse contact an individual has with the police via community incidents, word of mouth, and/or the media” [32]. The observed community-level effect of police violence supports a key assumption of our own analysis–that police violence can politically socialize Americans who are not personally subjected to it.

The preceding literature motivates our first hypothesis (H1): Jacob Blake’s shooting negatively affected perceptions of police for both white and Black respondents. We expect to see an effect among Black respondents in large part because Jacob Blake is Black; Morris and Shoub find stronger effects of police violence against Black victims on other Black residents’ political behavior [32]. Yet other recent work found more strongly diminished trust in police for white residents compared to Black residents [27, 33]. These apparently contradictory findings suggest that this theoretical space is still developing.

Sociologists have argued that criminal legal contact can shape individuals’ orientations towards a wide variety of public institutions, including but not limited to law enforcement. In her analysis of “system avoidance,” Brayne found that criminalized respondents were less likely to interact with institutions that might be perceived as surveilling due to a fear of re-arrest (e.g. hospitals, schools, banks), and the negative effect intensified alongside criminal legal contact (police stops, arrests, conviction, and incarceration) [13]. These findings were further substantiated by Remster and Kramer [34], and they track with the increasing severity of withdrawal observed in the political participation literature cited above (e.g. [29]). The system avoidance literature informs our second hypothesis (H2): *We expect the effect of Jacob Blake’s shooting to be more pronounced for residents who have had contact with the criminal justice system in the past (e.g., having been arrested, booked, jailed, or incarcerated)*.

Prior research leads us to believe that observed effects are likely to vary by age and gender. Exploring effect heterogeneity by age is important given that young adults and early middle-aged individuals play a key role in political activism and social movements [35]–and people who recently attended protests were much younger than the general population [36]. The presence of a strong narrative of racial injustice can compel members of oft-criminalized communities to participate in non-voting political behaviors such as signing petitions or volunteering for candidates [37, 38]. This theory is compatible with legal cynicism or legal estrangement to the extent that individual grievances are newly construed as signs of group-level inequality and accords with prior accounts of criminal legal contact catalyzing heightened political consciousness [17]. Protests are one site where such narratives could be cultivated and propagated. To the extent that protests may politically socialize participants [27, 32] we can propose our third hypothesis (H3): *The negative effect of Jacob Blake’s shooting on perceptions of police was more pronounced for younger Chicagoans*.

Evidence on the gendered experience of police encounters and police violence suggests that men, particularly those from minority groups, may be more likely to experience racial profiling, excessive use of force, or discriminatory treatment [15, 17, 39]. Such experiences can create a higher likelihood of mistrust and negative perceptions of the police. Yet women are also subjected to similarly alienating forms of police misconduct; for instance, young Black women’s views of police are colored by sexual harassment and sexual violence, and Black mothers consistently report fears that their children will experience police violence [9, 18, 40]. Accordingly, we formulate our fourth hypothesis (H4): *We expect trust in law enforcement to be similarly affected across Black men and women*.

To sum up, police legitimacy theory proposes that unjust interactions with police or high-profile police violence events experienced vicariously lead to reduced trust in police, thus leading to reduced legal compliance. The present study differs from past literature in that we estimate individual-level average causal effects and stratify our analyses along a wide range of individual characteristics.

## The events in Kenosha in August of 2020

The summer of 2020 in the United States was marked by racial tensions and social upheaval. The killing of George Floyd, a Black man, by a Minneapolis police officer on May 25, 2020 sparked nationwide protests. Floyd’s death became a symbol of entrenched systemic racism and police brutality. These racial tensions were further complicated by the COVID-19 pandemic, which disproportionately affected communities of color, exposing deep-seated disparities in healthcare, economic opportunities, and education.

It was against this backdrop that the shooting of Jacob Blake took place in Kenosha, Wisconsin. On August 23, 2020, officers responded to a domestic disturbance call involving Blake, a 29-year-old African-American who was unarmed. As Blake walked away from the officers, he was grabbed by his shirt and shot multiple times in the back by Officer Rusten Sheskey. Blake survived, but was left paralyzed from the waist down. The incident was captured on video and went viral, igniting widespread outrage and protests both in Kenosha and across the nation for 8 days following the shooting. The city declared a state of emergency, and the National Guard was deployed.

Amidst these protests, Kyle Rittenhouse, a white 17-year-old from Antioch, Illinois, crossed state lines armed with an AR-15-style rifle and arrived at Kenosha on August 24. Rittenhouse claimed he went to Kenosha to protect local businesses and provide medical aid, but on the night of August 25, 2020, during ongoing protests, Rittenhouse was involved in a series of confrontations that culminated in the shooting of three individuals.

This adds a layer of complexity to our study as it becomes difficult to disentangle the effects driven by the shooting of Jacob Blake from those driven by the Rittenhouse events. It could be that observed changes in trust in the week that followed Blake’s shooting resulted from the frustration of seeing police officers do nothing against an armed White man who shot three protesters. We will address these concerns in our empirical strategy by assessing the extent to which changes in trust in law enforcement were already visible in the two days before Rittenhouse shot three protesters.

## Methods

Identifying the causal effect of police violence on trust in the police is complicated by the fact that exposure to these events is not random. Black Americans are simultaneously more likely to be victims of lethal and aggressive police violence [15] and to show lower levels of trust in the police [41]. Although previous studies relying on survey data have shown that individuals with prior contact with the police report lower levels of trust in government [42], establishing a causal relationship between the experience of police violence and trust in law enforcement can be challenging. Correlational evidence may not accurately capture the sequence of events; an individual who reports low trust following a police violence event may have lost trust long before that event, suggesting reverse causality. Similarly, self-reported data often lack control over confounding variables such as racial identity and the experience of police violence that may influence both the experience of police violence and trust in the police. Without accounting for these variables, it is challenging to establish a direct causal relationship between police violence and trust.

In this study, we overcome these challenges by using a quasi-experimental design in which we leverage the timing of a police violence event relative to a wave of survey data collection. This approach is commonly called “unexpected event during survey design,” or UESD [43, 44]. Under the assumptions that underlie this research design, individuals surveyed before the shooting took place serve as plausible counterfactuals for individuals surveyed after the shooting. We assess the validity of this assumption in the next section.

To set up the UESD to answer our research questions, we link data from a survey that was fielded in Chicago around the time when the shooting of Jacob Blake took place (August 23, 2020). Our identification strategy relies on the exogenous timing of the shooting relative to when the survey was fielded. This allows us to compare the answers to questions about trust in the police across two sets of survey participants: those interviewed four weeks before the shooting took place and those interviewed four weeks after. We show that these two sets of respondents are statistically identical in terms of a large set of observed attributes.

### Data

Our analysis draws on Healthy Chicago Survey (HCS) data collected by the Chicago Department of Public Health between June 27 and December 9, 2020. The survey has been used to identify health concerns for each community in Chicago and to understand environmental, neighborhood, and social factors associated with health. It has been conducted annually since 2014, but the 2020 wave was the first iteration including questions about trust in police. In 2020, the survey’s format also shifted from a random-digit dial telephone survey to a self-administered, mixed-mode design wherein informants sampled by address could complete the survey online or by sending in a pencil-and-paper form.

The sampling frame in the HCS survey ensured that the data were representative of the Chicago population. RTI International, the organization in charge of designing and implementing the 2020 HCS survey, geocoded all Chicago postal addresses (N = 1,201,979) and nested them from the 77 community areas that divide the city of Chicago. From that universe of addresses, RTI targeted a minimum of 35 survey completes within each of the 77 community areas and a total of 4,500 survey completes overall. We refer readers to the 2020 Healthy Chicago Survey Methodology Report for additional details on the design and implementation of the survey [45]. The HCS sample includes 4,474 Chicago residents who are at least 18 years old. Due to its large sample size and city-wide coverage, a number of studies have used the HCS survey to study health outcomes in Chicago ([46, 47]).

We focus on the subset of 584 Black and 939 white respondents interviewed during the eight weeks that surrounded the shooting. During that same period, only 389 Hispanic Chicagoans were surveyed, leaving us statistically under-powered to test all hypotheses for this group. The duration of this window is informed by prior studies using similar windows [48]. We focus on Black and white respondents due to statistical power limitations that prevent us from carrying out subgroup analyses for respondents of other racial and ethnic backgrounds. The constraints of the UESD design may raise concerns about the representativeness of our analytical sample. We evaluate these concerns by assessing differences across the pool of Black and white HCS survey respondents that made it to our analytical sample and Black and white HCS survey respondents that were excluded because they happened to be interviewed outside the four weeks before and after the shooting.

Ethics approval (i.e., IRB) for this study was not necessary because we are analyzing non-identifiable, publicly available survey data. All of the analyses are on secondary data, and there is no linkage of individual-level survey data to any other datasets.

The first column in Table 1 reports descriptive statistics for the subsample of Black and white respondents included in our UESD analyses. The second column shows descriptive statistics for Black and white respondents whom we excluded due to their survey response date being prior to the four weeks before or posterior to the four weeks after the shooting. We report proportions in the different categories that define race (Black and white); gender (male, female, and other gender identities); age (aged 18–29, 30–44, 45–64, and 65 and above); educational attainment (less than high school, high school diploma, and bachelor’s degree or more); home ownership status; employment status; having lived in the neighborhood for more than 5 years; having ever been arrested, booked, or jailed; and having any children. We find that our UESD analytical sample of 1,523 survey participants is almost identical to the 1,425 survey respondents that we excluded from the UESD analyses. One pattern that stands out from Table 1 is that the HCS data are not fully representative of the Chicago population. This is apparent by looking at the gender composition of the included and excluded subsamples, which are both a third male and two-thirds female. While RTI International randomly selected addresses to be contacted in each of the 77 community areas, they did not impose any restrictions on which adult member of the household would respond to the questionnaire. It is important to note, however, that demographic composition discrepancies do not pose any threats to the internal validity of our findings. 62% of respondents in our analytical sample are white and 38% are Black; 37% are male, 62% are female, and 1% identify with another gender; 16% have ages between 18 and 29, 26% have ages between 30 and 44, 31% have ages between 45 and 64, and 27% have ages 65 and above. When compared to the race, gender, and age composition of the rest of the HCS data, we don’t observe substantial differences except for the somewhat larger share of individuals aged 65 and above in our sample. Moving to educational attainment, we see that 3% of respondents in our sample did not complete high school, 13% ended their education with a high school degree only, 24% have completed some years of college education, and 58% have a college degree or more. These proportions are almost identical in the subsample of excluded HCS survey respondents. The last set of covariates show that 45% of our respondents are homeowners, 56% were employed at the time of the survey, 59% of them had lived in the neighborhood for at least 5 years, and 12% had been arrested, booked, or charged at some point in the past.

**Table 1:** Demographic characteristics of Black and white survey respondents in the included and excluded samples.

	Respondents included in the UESD design (N = 1,523)	Respondents excluded from the UESD design (N = 1,425)
White	0.44	0.40
Black	0.27	0.27
Male	0.37	0.36
Female	0.62	0.63
Other gender	0.01	0.00
Age 18–29	0.18	0.17
Age 30–44	0.29	0.32
Age 45–64	0.29	0.33
Age above 65	0.24	0.18
Less HS	0.06	0.06
HS	0.14	0.15
Some college	0.25	0.27
Bachelor’s degree	0.54	0.51
Homeowner	0.43	0.45
Employed	0.55	0.58
More than 5y in neighborhood	0.60	0.63
Ever arrested, booked, or jailed	0.10	0.10

Data are from the 2020 Healthy Chicago Survey. The included sample includes 584 Black respondents and 939 white respondents interviewed in the four weeks prior and four weeks after the shooting of Jacob Blake; the excluded sample includes 570 Black respondents and 855 white respondents interviewed outside the four weeks prior and four weeks after the shooting of Jacob Blake. The first column reports proportions for the corresponding covariates among those in the included sample; and the second column shows proportions for those the excluded sample.

**Fig 1:**
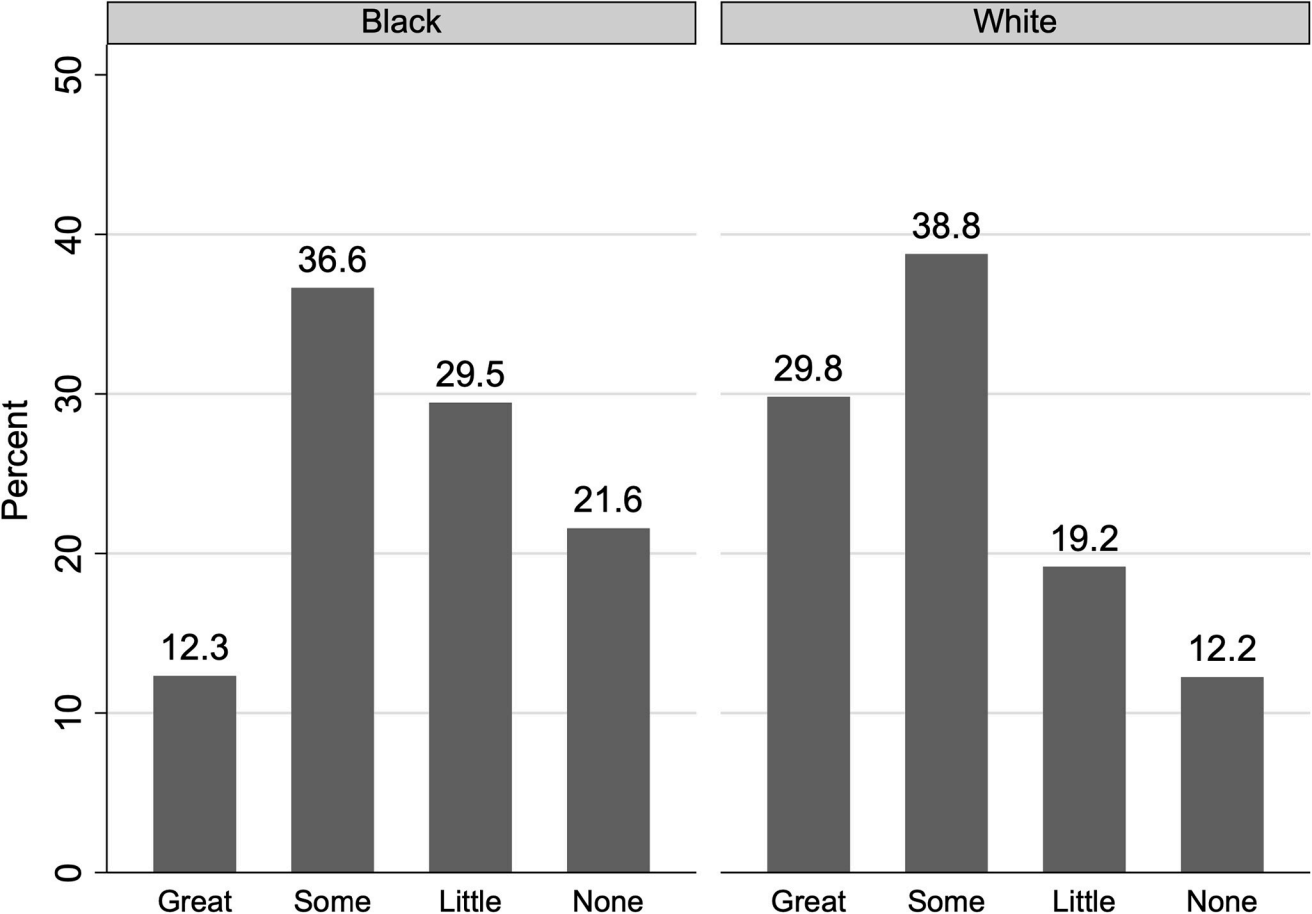
Trust in law enforcement among the Black and white respondents included in the analyses. Data are from the 2020 Healthy Chicago Survey. The sample includes 584 Black respondents and 939 white respondents interviewed in the four weeks prior and four weeks after the shooting of Jacob Blake. The survey question underlying the graphs is *“To what extent do you trust your law enforcement agency?”*.

The outcome measures in our analysis of HCS data reflect respondents’ trust in local police. We construct the dependent variable from respondents’ answers to these this question: *“To what extent do you trust your law enforcement agency?”* The possible responses to this question include *“a great extent, somewhat, a little, not at all.”* We code these variables as binary, taking on value 1 for those that responded *“a great extent”* or *“somewhat”* and 0 for the rest. Fig 1 shows the distribution of responses to the questions that we use as outcomes regarding trust in police. We find that 49 percent of Black respondents express great or some trust in the police, with this percentage increasing to 69 for whites. In S1 Table in S1 File, we show regression-adjusted differences in trust across Black and White respondents.

**Fig 2:**
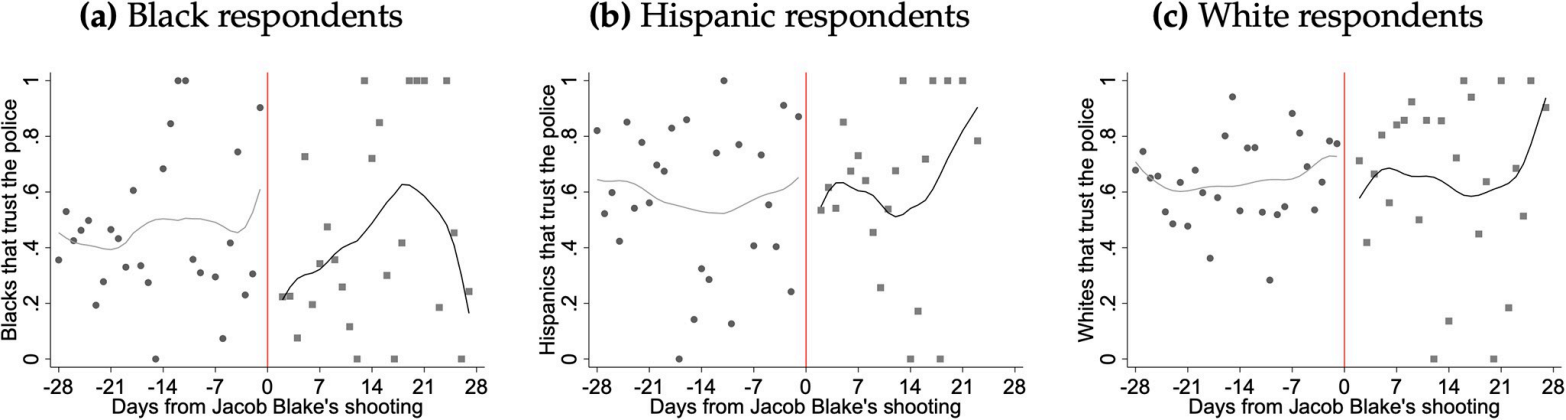
City-wide share of survey participants that reported trust in law enforcement in the days before and after Blake’s shooting.

Fig 2 begins to examine changes in our outcome of interest in a temporal way. It presents the city-wide share of survey participants that reported trust in law enforcement in the days before and after Jacob Blake’s shooting, with separate panels for different racial groups: Black respondents (Panel a), Hispanic respondents (Panel b), and White respondents (Panel c). The horizontal axis of each graph represents the days from Jacob Blake’s shooting, with the day of the shooting denoted by a vertical red line at day 0. The vertical axis measures the share of respondents that trust the police, ranging from 0 to 1. Each panel’s data is represented by individual dots for each day, with a smoothed line to indicate the overall trend over time. In Panel (a), trust among Black respondents shows a noticeable decline beginning shortly before the shooting and reaching its nadir in the days immediately following the incident. The pattern indicates a sharp drop in trust in the day after the shooting, which then fluctuates but stays below the pre-incident levels for the observed period. In Panel (b), Hispanic respondents’ trust appears to fluctuate widely before and after the shooting, with a less pronounced dip in trust immediately after the shooting. Panel (c) shows the trust levels among White respondents, which does not exhibit a significant change at the time of the shooting but does show some fluctuation in the days following. Overall, the level of trust among white respondents remains relatively stable compared to Black respondents.

S2 Table in S1 File shows the proportion of respondents who showed some or a great deal of trust in the police for the different subgroups for whom we estimate the impact of the shooting (i.e., race, race by age, race by criminal justice contact, and race by gender). The baseline levels of trust among the group of respondents surveyed in the four weeks before the shooting will be useful to interpret the relative size of the causal effects that our models estimate. We also show the number of respondents in each subgroup. For the analyses of Black respondents, our data include 430 respondents in the four weeks prior and 154 respondents in the four weeks after. The analyses of whites include 730 respondents in the four weeks prior and 209 respondents in the four weeks after. The sample shrinks substantially as we further stratify by other attributes. For example, for the analyses of respondents with prior contact with the police, we have 75 Black respondents in the four weeks prior and 26 in the four weeks after. For white respondents with prior contact with the police, we are left with 49 in the four weeks prior and 27 in the four weeks after. One clear pattern in S2 Table in S1 File is that there are fewer observations in the group that responded four weeks after the shooting. Across all subgroups analyses, individuals in post-shooting group are approximately evenly distributed across the four weeks that followed the event. This is a feature of the data rather than a bug. Looking at S1 Fig in S1 File, it is clear that the number of daily survey responses was generally declining over time. This downward trend in survey responses is “smooth” and does not show any discontinuity around the date of Blake’s shooting, thus strengthening the validity of our UESD design. What is relevant is that the composition of the before and after groups is generally balanced across covariates, something that we have shown in Table 2. The modeling approach that we describe below is designed to deal with these features of the data and their related sample size concerns.

**Table 2:** Demographic characteristics of survey respondents 4 weeks before and 4 weeks after Jacob Blake’s shooting.

	Mean 4w before	Mean 4w after	Diff. After-Before	P-val Diff.
White	0.48	0.46	-0.02	0.44
Black	0.25	0.30	0.05	0.05
Male	0.37	0.39	0.02	0.44
Female	0.63	0.61	-0.02	0.47
Other gender	0.01	0.00	-0.00	0.68
Age 18–29	0.19	0.19	-0.00	1.00
Age 30–44	0.32	0.30	-0.02	0.44
Age 45–64	0.29	0.30	0.02	0.49
Age above 65	0.20	0.21	0.00	0.93
Less HS	0.04	0.06	0.02	0.19
HS	0.12	0.15	0.03	0.08
Some college	0.25	0.26	0.01	0.68
Bachelor’s degree	0.59	0.53	-0.06	0.03
Homeowner	0.44	0.43	-0.02	0.51
Employed	0.59	0.60	0.01	0.83
More than 5y in neighborhood	0.57	0.59	0.01	0.61
Ever arrested, booked, or jailed	0.10	0.13	0.03	0.08

Data are from the 2020 Healthy Chicago Survey. The sample includes 584 Black respondents and 939 white respondents interviewed in the four weeks prior and four weeks after the shooting of Jacob Blake. The first column reports the mean for the corresponding covariates among those interviewed 4 weeks before the police violence event; the second column shows the mean for those interviewed 4 weeks after the police violence event; the third column shows the difference in means for these two groups; and the fourth column reports the p-value from a test of significance for the difference in means shown in the third column.

### Quasi-experimental design

We propose a UESD design that allows us to compare responses from HCS survey participants interviewed four weeks before and after the police violence events occurred. We construct a control group by pooling survey responses submitted in the 4-week period preceding the shooting. This larger control group is compared against four treatment groups, each corresponding to a one-week period in the four weeks following the shooting (i.e. August 24 to August 31 is the first treatment group). The analytical sample includes 584 Black respondents and 939 white respondents. S2 Table in S1 File shows the sample size for the treatment and control groups in the different subgroup analyses that we run.

The UESD design exploits survey response timing, which makes it conceptually similar to the regression discontinuity and interrupted time series designs. Two assumptions allowing identification of causal effects using UESD are excludability, or that the timing of the interview only affects the outcome through the treatment; and ignorability, or that the timing of each interview is independent from the potential outcomes [43].

In order to assess whether the ignorability assumption is satisfied, we conduct a covariate balance test and show that the set of survey respondents interviewed four weeks before the shooting is statistically equivalent to the set of survey respondents interviewed four weeks afterward, resembling the balance that one would expect if exposure to police violence had been randomly assigned. The empirical argument that informs our UESD design is that the timing of Jacob Blake’s shooting is exogenous with respect to the timing of when the HCS surveys were completed. This assumption could be violated if survey participants were contacted in a manner that prioritized respondents living in certain community areas over others. Having a larger share of Black respondents in the group interviewed after the shooting could confound any observed differences in trust after the shooting given that Black residents are generally less likely to trust law enforcement. Similarly, our ability to identify the causal effect of police violence on trust would be compromised if the occurrence of the shooting was what encouraged some survey participants to respond to the survey. Because the survey was self-administered, participants had the freedom to respond to the survey within a specified time frame. The selected survey participants received an invitation letter containing instructions to access the web survey and personalized login credentials. A week later, a reminder postcard was mailed to all addresses encouraging participation online. Two weeks later, non-respondents received a full paper questionnaire packet.

The balance assessment in Table 2 shows proportions for the group of respondents from four weeks before and after Blake’s shooting (first and second columns). To evaluate any imbalances, we perform t-tests for the differences in proportions across the two groups, reporting the size of the difference (third column) and the p-value from the t-test for the difference in proportions (fourth column). For most attributes, the balance assessment test reveals no systematic differences across the pool of respondents—differences in proportions are generally small and statistically non-significant at the 5% level. However, the pool interviewed after the shooting skews Black and has fewer college-educated respondents. It also includes a larger share of respondents with prior contact with the criminal justice system. Looking at patterns of survey response across community areas, we don’t observe any systematic differences in the order in which surveys from different areas were submitted. However, if community areas had been surveyed in sequential order, we could have ended with some demographic groups being more represented in the before or after groups, potentially skewing the results and introducing bias into our analysis. This could be particularly significant if some community areas have residents that are wealthier or have a higher proportion of a given racial group, which we know is the case in Chicago and most American cities.

So the differences observed in Table 2 appear to result from Black, lower-SES respondents feeling more compelled to complete the survey after the shooting. We mitigate concerns about possible compositional changes by running and reporting models with and without controls. Similarly, because we estimate models by race and criminal justice contact status (and the combination of the two), the slight imbalance seen in Table 2 becomes less problematic. Although we cannot be certain that our treatment and control groups do not differ across unobserved attributes, seeing no differences in the comprehensive list of covariates shown in Table 2 give us confidence that the ignorability assumption is not violated.

With respect to the excludability assumption, S1 Fig in S1 File shows that the frequency of HCS survey completion was not significantly altered by Jacob Blake’s shooting. A large discontinuity in responses to the survey following the shooting could suggest selection or sorting on the basis of when the survey was conducted. Fortunately, we observe no such discontinuity. Similarly, it would be cause for concern if the unexpected event appeared to affect responses to theoretically unrelated questions. Our falsification test found no evidence of this (see S6 Fig in S1 File).

The empirical estimand in our analyses, then, is the difference in perceptions of police between survey respondents who completed surveys in the weeks preceding and weeks following a widely-known police violence incident. The estimating equation that the UESD approach relies on is as follows:

$$\begin{array}{c} Y_{i}=\alpha +\sum_{j=4}^{J}\delta^{j}{\text{Post}}_{i}^{j}+\sum_{j=1}^{p}X_{i}\beta_{j}+\epsilon_{i} \end{array}$$ (1)

In Eq (1), *Y*_*i*_ is a binary indicator that takes on value 1 if the respondent expressed great or some trust in law enforcement, and 0 otherwise. The term $\sum_{j=4}^{J}{\text{Post}}_{i}^{j}$ represents a set of binary indicators capturing whether respondent *i* was surveyed in weeks 1, 2, 3, or 4 after the shooting. The corresponding coefficients, $\sum_{j=4}^{J}\delta^{j}$, are the estimated effects for each of these indicators. The term $\sum_{j=1}^{p}X_{i}$ is a vector of respondent-level controls, and *ϵ*_*i*_ is an idiosyncratic error term. The set of controls in $\sum_{j=1}^{p}X_{i}$ are those listed in Table 2 (gender; age; educational attainment; homeownership; employment; years of residence in the neighborhood; and arrest record). The balance test shown in Table 2 indicates that most of the observed covariates are balanced across the before and after groups. For the attributes that show statistically significant differences (Black, education level, and police contact), the relative size of these differences is small in magnitude. Including the controls in $\sum_{j=1}^{p}X_{i}$ should alleviate concerns about the potential impact of any differences across the before and after groups. For the sake of transparency, we report estimates with and without controls for all our analyses. Standard errors are robust to heteroskedasticity. We test our four hypotheses by estimating separate models by race (Black and white), race by criminal justice contact, race by age, and race by gender. We report results from these subgroup analyses in a series of coefficient plots that show temporal changes in the effects week by week over a four-week period.

This model specification allows us to assess the duration of the decline in trust following the shooting, as captured by the $\sum_{j=4}^{J}\delta^{j}$ coefficients. It also enables us to utilize the full set of respondents surveyed four weeks before and after the incident, rather than estimating separate models for each individual week within the four weeks post-shooting. This is important because despite being evenly distributed across weeks, individuals surveyed in the four weeks after the shooting represent a smaller group than those surveyed in the four weeks before (see S2 Table in S1 File). As discussed before, this is expected given the smooth but downward trend in the rate at which surveys were conducted (see S1 Fig in S1 File). By estimating all weekly effects simultaneously and including the set of controls $\sum_{j=1}^{p}X_{i}$, we improve statistical power and address sample size concerns in some of the subgroup analyses needed to test our hypotheses [49].

## Results

**Fig 3:**
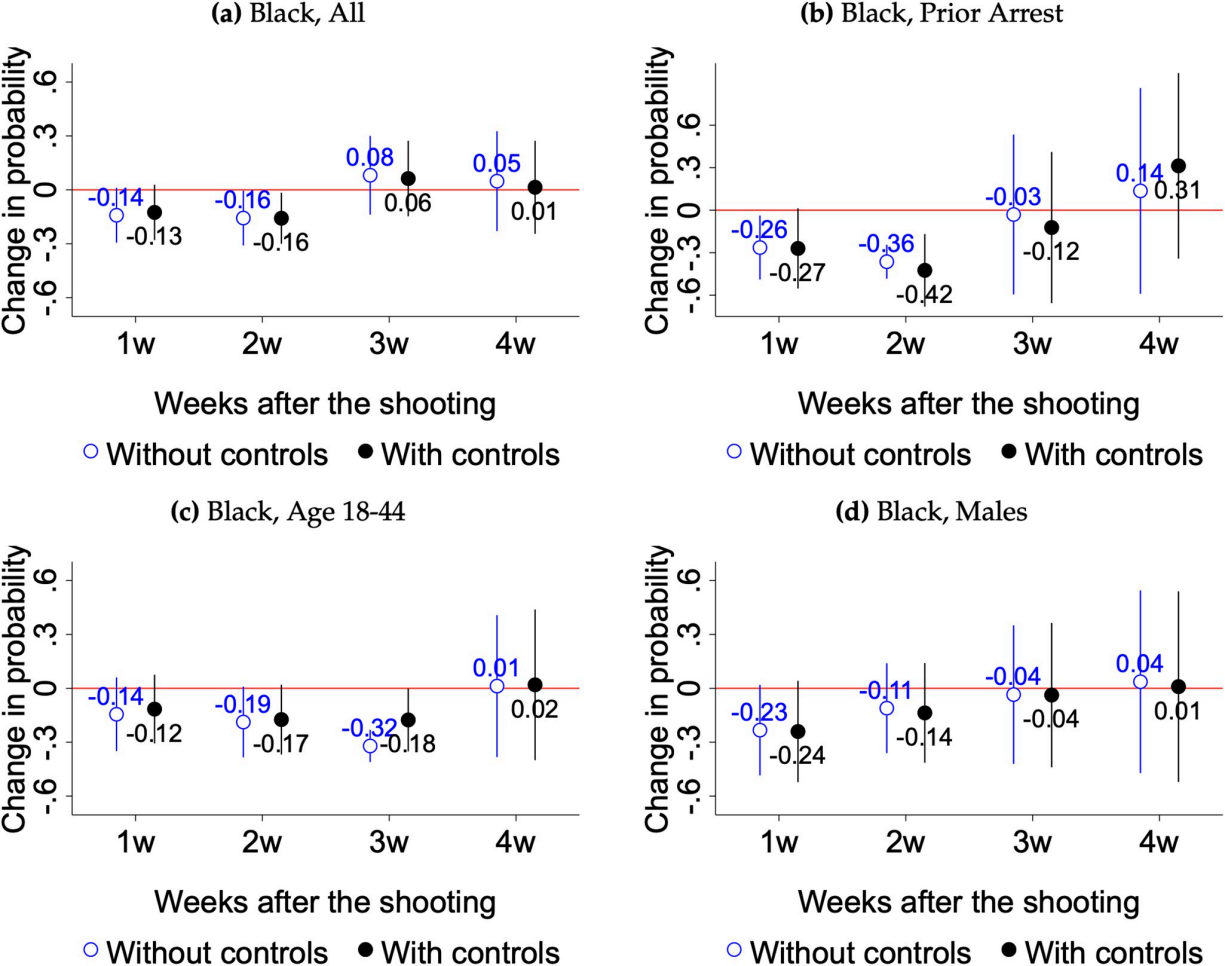
Changes in trust in law enforcement after Jacob Blake’s shooting among Black respondents. Data are from the 2020 HCS survey. The survey question used to create the measure of trust in law enforcement is *“To what extent do you trust your law enforcement agency?”* We code answers to this question as binary, taking on value 1 for those that responded *“a great extent”* or *“somewhat”* and 0 for the rest. Each pair of point estimates shows the change in the probability of expressing trust for those surveyed one, two, three, and four weeks after Jacob Blake’s shooting, relative to the pool of survey respondents interviewed in the four weeks prior. Within each pair of estimates, we show results with and without the set of controls listed in Table 2. Confidence intervals are at the 95% level with standard errors robust to heteroskedasticity.

**Fig 4:**
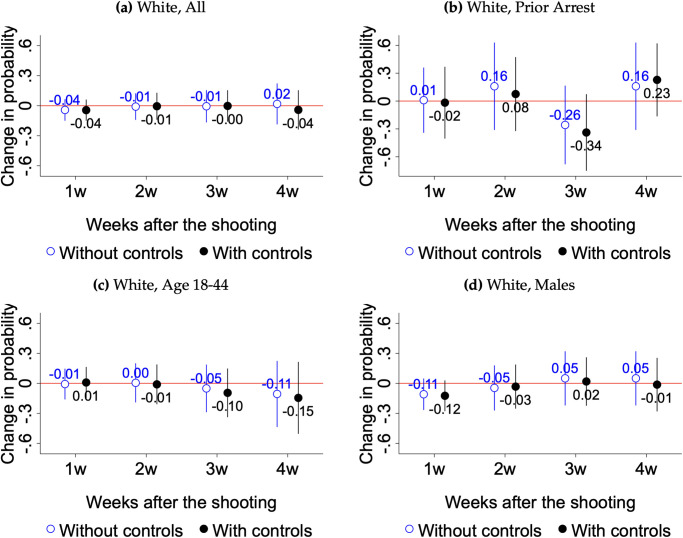
Changes in trust in law enforcement after Jacob Blake’s shooting among White respondents. Data are from the 2020 HCS survey. The survey question used to create the measure of trust in law enforcement is *“To what extent do you trust your law enforcement agency?”* We code answers to this question as binary, taking on value 1 for those that responded *“a great extent”* or *“somewhat”* and 0 for the rest. Each pair of point estimates shows the change in the probability of expressing trust for those surveyed one, two, three, and four weeks after Jacob Blake’s shooting, relative to the pool of survey respondents interviewed in the four weeks prior. Within each pair of estimates, we show results with and without the set of controls listed in Table 2. Confidence intervals are at the 95% level with standard errors robust to heteroskedasticity.

We report the main findings in Figs 3 and 4 and supplementary ones in S3 to S6 Figs in S1 File. As stated above, we lack sufficient power to run subgroup analyses by age, gender and criminal justice contact status among Hispanics, Asians, and respondents of other racial groups. Nonetheless, we report estimated impacts for all Hispanics in S3 Fig in S1 File. Each pair of point estimates in the coefficient plots shows the change in the probability of expressing trust for those surveyed one, two, three, and four weeks after Jacob Blake’s shooting, relative to the pool of survey respondents interviewed in the four weeks prior. Within each pair of estimates, we show results with and without the set of controls listed in Table 2.

Our results provide varying degrees of support for our hypotheses. The clearest evidence is that in support of H1, H2 and H3: the shooting of Jacob Blake led to a decline in trust in law enforcement among the Black community driven by younger cohorts and by individuals who had previously been arrested or incarcerated. Notably, the baseline level of reported trust was lower among these subgroups of Black respondents—49 percent of all Black respondents in the control group said that they trusted police before Jacob Blake’s shooting, whereas only 32 percent of Black respondents aged 18–44 and 36 percent of Black respondents with prior arrests reported that they trusted police.

Focusing on H1, Figs 3a and 4a show results when all Black and white respondents are evaluated as a group. We find a 13 to 16 percentage point decline in trust in police among all Black respondents who completed surveys in the first and second weeks after Jacob Blake was shot. These declines are relative to the control group’s average of 49 percentage points, as shown in S2 Table in S1 File. For whites, we do not observe any substantial changes in police trust after Blake’s shooting. H1 is the only hypothesis that we have sufficient power to test among Hispanics. Results in S3 Fig in S1 File show no effect for this set of respondents.

To test H2, Figs 3b and 4b limit the analysis to respondents who reported previously being arrested, jailed, or imprisoned. We find a significant decline in reported trust in law enforcement among Black respondents: 27 and 42 percentage points in the first and second weeks, relative to a baseline of 36 percentage points. In S4 Fig in S1 File, we report smaller declines in trust in law enforcement among Black respondents who have never been arrested, jailed, or imprisoned. These patterns are consistent with H2. We don’t find any changes in trust among white respondents in this subgroup.

With respect to H3, Figs 3c and 4c show a substantial decline in trust in police among Black respondents aged 18–44. We observe declines ranging from 12 to 18 percent across the first three weeks (from a baseline of 32 percentage points). When we compare these estimates to those among Black respondents that are older than 44 (S4 Fig in S1 File), we observe somewhat smaller declines in trust that lasted only for two weeks. While the coefficients in the two weeks following the shooting are not statistically different across the two age groups, the effect for the younger cohort increases substantially in the third week whereas the effect for the older cohort disappears. We interpret this as evidence in support of H3. As before, we find no effect among white respondents in these two age groups.

With respect to gender, we find suggestive evidence in support of H4 in Figs 3d and 4d. Black men are the group that experienced more substantial declines in trust in law enforcement in the week after Blake’s shooting: a 24-percentage-point drop from a baseline of 43 percentage points. This effect, however, is only significant at the 10 percent level. As shown in S4 Fig in S1 File, Black women exhibit smaller, statistically non-significant effects in the second week following the shooting.

### Separating the Kyle Rittenhouse events

The results presented thus far indicate that Black respondents report lower trust in law enforcement in response to police violence events. One valid concern about our empirical approach is that the effect of Blake’s shooting could be influenced by the effect of the events in the days that followed. Blake was shot in Kenosha on August 23, 2020, and on the night of August 25, Kyle Rittenhouse shot three protesters in Kenosha, with both events receiving extensive news coverage at the national level. It is plausible to think that Black survey respondents lost trust in law enforcement not only because a cop shot another unarmed Black man, but because Kyle Rittenhouse (a white man) was able to attend a protest armed with an AR-15-style rifle, shoot three individuals, and return back to his hometown without being stopped or arrested by the police (he turned himself in on August 26). With a large enough sample, we could run an analysis where we model changes in trust in one-day intervals after the shooting, allowing us to observe daily changes in trust from August 23 to August 25. If the effect is driven by Blake’s shooting, we should see a decline in trust in the days when Kyle Rittenhouse had not yet arrived in Kenosha and shot anyone.

Seeing trust declines in the two days after Blake’s shooting would support our theory that police violence increases feelings of mistrust among the Black population. This is what we find in S5 Fig in S1 File. The coefficient plot shows changes in trust in two-day intervals during the six days that followed Blake’s shooting. The control group remains the set of survey participants who responded to the survey in the four weeks prior to Blake’s shooting. These models include the same set of controls that we use in models that correspond to Figs 3 and 4. We estimate these models for the two groups for which we found the clearest effects in the models discussed above—all Black respondents and Black respondents with prior contact with the police or the criminal justice system. The coefficients that help us separate the Blake effect from the Rittenhouse effect are those estimating changes in the Aug 24–25 window. Since Rittenhouse began his shooting spree at 11:48pm on August 25, survey responses submitted on August 25 had not yet been influenced by the Rittenhouse events. We find that the trend seen in Fig 3 was set in the immediate aftermath of Blake’s shooting and before Rittenhouse shot anyone. On August 24–25, we see a 26-percentage-point decline in trust in law enforcement among all Black respondents and a 52-percentage-point decline in trust in law enforcement among Black respondents who reported prior contact with the criminal justice system. Both of these estimates are statistically significant at the 5% level. Seeing that levels of trust dropped within 48 hours of Blake’s shooting increases our confidence that the Rittenhouse events are not driving our core findings.

### Alternative explanations and falsification test results

Blake’s shooting and the resultant protests unfolded during the most intense days of the COVID-19 pandemic. A change in the frequency and nature of police-civilian interactions in the local context could have influenced perceptions of trust. Evidence from Houston shows that reactive police activities (e.g., deployments of special units) significantly decreased during the pandemic, but proactive patrols significantly increased [50]. To assess the possibility that the decline in trust that our models identify is a result of Chicagoans’ perceptions of city-wide changes in the frequency of police activity, we look at discontinuities in arrest patterns in the weeks before and after Blake’s shooting. S2 Fig in S1 File shows the number of arrests in Chicago in the weeks before and after Jacob Blake’s killing, broken down by racial categories: Black arrests (Panel a), Hispanic arrests (Panel b), and white arrests (Panel c). The horizontal axis on each graph spans the weeks surrounding Jacob Blake’s shooting, with the event marked by a vertical red line at week 0. The vertical axis represents the number of arrests per week for each group. We don’t see any noticeable discontinuity in the number of each type of arrest in the weeks surrounding Jacob Blake’s shooting.

Additionally, it could be the case that local COVID-19 policies had some effect on perceptions of police (i.e., if police were enforcing stay-at-home orders). A search of Chicago COVID-19 policies and local news articles during the survey period provides little evidence suggesting that COVID-19 policies confound our analysis. The state of Illinois phased out stay-at-home orders in late May, before the 2020 HCS survey was fielded. Although an additional stay-at-home advisory was issued towards the end of the HCS survey period (effective November 16, 2020), this was less stringent than a stay-at-home order and also falls outside the temporal window for the analytical sample used in this study.

In S6 Fig in S1 File, we assess whether Jacob Blake’s shooting changed an outcome that should not have been affected by the shooting. This test is recommended to generate suggestive evidence to support the excludability assumption [43]. Unfortunately, the HCS data don’t include many questions related to behavioral outcomes that could be used for this test. Most of the questions ask respondents to report on health-related issues retroactively over long periods of time (e.g., having ever been diagnosed with hypertension), rendering them unsuitable for the falsification test. The only question that speaks to a behavioral outcome that could have changed over a short period is one on soda consumption habits. HCS participants were asked “During the past 30 days, how many regular soda or pop or other sweetened drinks like sweetened iced tea, sports drinks, fruit punch or other fruit-flavored drinks have you had per day?” We use this question to test whether the shooting changed the probability of consuming more than one soda drink per day. We find no evidence of any changes in soda consumption in the aftermath of the shooting.

## Discussion

While prior research has thoroughly probed the descriptive relationship between criminalization and perceptions of police, few prior studies credibly identify individual-level causal effects. We examine whether these effects differ by age, gender, racial identity, or prior exposure to criminalization. While some recent work has examined effect heterogeneity according to e.g. partisan identification and media consumption habits [27], our study builds on gaps in existing literature by testing effects among various demographic subgroups and gauging their durability over time.

We find substantial, yet short-lived declines in perceptions of law enforcement among Black Chicago residents following the shooting of Jacob Blake. These effects were particularly pronounced among younger cohorts and respondents who reported prior contact with the criminal legal system. Unlike prior work, we do not find particularly strong evidence of a stronger effect for Black men compared to Black women. We also find that the decline in trust that we report in our core findings began in the immediate aftermath of Blake’s shooting—before Rittenhouse arrived in Kenosha and shot three protesters.

We believe that three of our four hypotheses pass a ‘hard test’: we saw declines in trust in police among Black Chicagoans whose quasi-experimental counterparts already reported very low levels of trust before Jacob Blake’s shooting. These respondents had likely been exposed to the police murder of George Floyd a few months earlier, which was a similarly salient continuing national news story. If we think about the Black Chicagoans in our study as subjects of many prior, analogous “treatments,” the fact that we observed any effect at all is notable.

We acknowledge that some features of this police violence incident (the Kyle Rittenhouse events and potential confounding related to the the COVID-19 pandemic) may initially give readers reason to doubt the viability of our identification strategy. We endeavoured to address these concerns through a series of robustness tests which ultimately increased our confidence in the analysis and findings.

Another potential threat to our identification strategy is noncompliance, i.e., some survey respondents may not have been aware of the police violence event [43]. If this were the case, our estimates could be interpreted as “intent-to-treat” (ITT) rather than the average treatment effect (ATE). That being said, Blake’s shooting was highly salient both in Chicago and nationwide and was covered by all major newspapers, TV, and radio channels for several days.

One limitation of the UESD design (in general) is that unique events may limit the generalizability of findings. Unfortunately, police violence is a relatively frequent event in America: according to the nonprofit research organization Mapping Police Violence, at least 1,176 people were killed by police in 2022. Granted, most of these killings do not reach the same level of public awareness as e.g. George Floyd, but police violence is still a regular phenomenon, and media coverage of police violence has increased over time [27].

We are also unable to probe certain causal mechanisms driving observed effects. For example, Reny and Newman [27] tests self-reported media consumption and geographical proximity to George Floyd protests. It could be the case that our observed effects were driven in part by news outlets’ amplification of Jacob Blake’s shooting or viral social media posts [51, 52]. Although it is true that news coverage of police violence has increased following the rise of the Black Lives Matter movement [53], we find implausible that our observed effects primarily result from selective media amplification of police violence incidents. Research on social media usage shows that individuals who do not intentionally use social media for news but encounter it incidentally on platforms like Facebook, YouTube, and Twitter tend to engage with a wider array of online news sources compared to those who do not use social media at all [54]. This incidental exposure effect is notably stronger among younger users and those with a low interest in the news. This suggests that, independently of how traditional news outlets covered Jacob Blake’s shooting, the occurrence of such an event likely reached a broad segment of the population.

Furthermore, past research suggests that individuals who have previously been arrested (one of the groups for which we observe the strongest effects) are less likely to have their views influenced by crime news coverage [55]. Additionally, even if fewer news stories about the shooting were published, it seems possible that viral social media posts would have ensured that the survey respondents were informed about the shooting. Further research is required to better formulate a complete account of legal cynicism via police violence. Our design also cannot differentiate between the effect of police violence itself and the effect of ensuing protests. Yet there would be no police brutality protests without police brutality.

Finally, survey responses may be an imperfect proxy for actual beliefs and behaviors, and particularly with respect to law enforcement (as highlighted by [7]). This is particularly true when measuring interactions with the police, as surveys can be less reliable in capturing genuine experiences and sentiments [56]. While some might express a reluctance to cooperate with the police in surveys, their actions might indicate otherwise. This divergence between stated attitudes and actual behaviors underscores the potential pitfalls of relying on survey data alone. Yet for the specific question examined in this paper—whether police violence affects trust in police (rather than e.g. willingness to report a crime)—these potential shortcomings are likely less salient.

Moving away from the limitations of our study, the fact that we observed substantial effects only among Black respondents is somewhat puzzling because recent, similar studies have found different results. In the wake of George Floyd’s murder, Reny and Newman found that changes in white Americans’ attitudes towards police were larger than changes among Black Americans [27]. Anoll and coauthors found that white survey respondents’ attitudes towards law enforcement were much more strongly associated with recent criminal legal contact compared to Black men [33]. Yet we observed a much stronger effect for Black respondents–even when we limited the sample to only those who reported prior police contact. Speculating beyond the survey data, it could be the case that white supremacist organizing in defense of Kyle Rittenhouse following Jacob Blake’s shooting by police influenced the average effect we observed for white respondents. The chronology of police violence events in 2020 may also be explanatory–perhaps changes in white attitudes towards police had already reached a ‘ceiling’ following George Floyd protests in the spring and summer.

The idea that our target population was “pre-treated” via exposure to previous police violence incidents (e.g. George Floyd) may also help to explain why most of the effects we observe here appear to be short-lived. To the extent that acquiring new information about a police violence incident constitutes ‘learning’ about one’s own relationship to police or government more broadly, Black residents have ‘less to learn’ due to their disproportionate historical exposure to policing and incarceration (at the personal, proximal, and community levels). Some recent work has found similar temporal bounds for related treatment effects, i.e., Black residents who were ticketed closer to the date of an election were less likely to vote compared to voters who were ticketed further away from the election date [31]. It could have been the case that Black respondents experienced a powerful short-term reaction (i.e., “anticipatory stress of police brutality,” per Alang and coauthors [57]) when hearing about a new police violence incident, and the salience faded as the incident was incorporated into pre-existing knowledge and narratives of group-level exposure to criminalization.

We also note with interest that we observed more pronounced negative effects among Black men than Black women, which may be seen as contrary to the predictions of prior scholarship. It may be the case that Black men responded more strongly to Jacob Blake’s shooting since the specific context of the incident corresponded more directly to the types of police violence or misconduct most commonly experienced by men rather than women; the vast majority of American police shooting victims are men.

To the extent that legal cynicism might mediate police violence and e.g. political participation or system avoidance, the direction of any subsequent causal effect is not a given. For example, while personal exposure to criminalization tends to decrease the likelihood that an individual will vote [30, 31], community-level indirect exposure can increase voter turnout [32]. Although Brantingham and coauthors found stable crime reporting trends in the wake of George Floyd protests [58], this may reflect an awareness that police were the only available option that residents could turn to in response to e.g. interpersonal violence. Further research is required to determine how legal cynicism affects participation in civic life across a variety of contexts and for different groups of Americans. These effects could also plausibly shift over time, as Americans are exposed to new “injustice narratives” and nascent protest movements.

Taken together, our findings enhance our understanding of the ways in which racial identity and the lived experience of criminalization affect perceptions of police. In addition to prior literature suggesting that declining trust in police could lead to e.g. lower rates of crime reporting, our study suggests that police violence drives legal cynicism among Black youth, plausibly shaping a much wider range of activities of interest.

## Supporting information

S1 File Appendix containing additional figures and tables. (PDF)
