# Supplementary material for: Police violence reduces trust in the police among Black residents
Source: PLoS One. 2024 Sep 11;19(9):e0308487. doi: 10.1371/journal.pone.0308487 (PMC11389903; doi:10.1371/journal.pone.0308487)
Supplement: S1 File — (PDF) [file pone.0308487.s001.pdf]

Supporting information

.

Figure S1: Number of surveys conducted in the days before and after Blake's killing

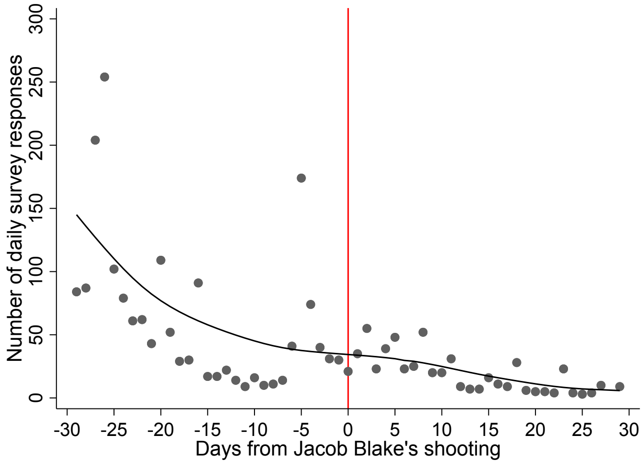

Figure S2: Number of arrests in Chicago in the weeks before and after Blake's killing

(a) Black arrests

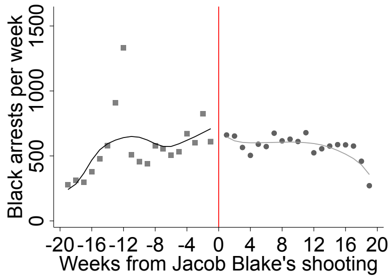

(b) Hispanic arrests

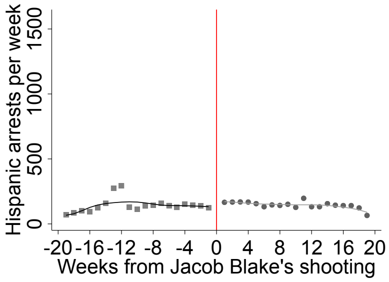

(c) White arrests

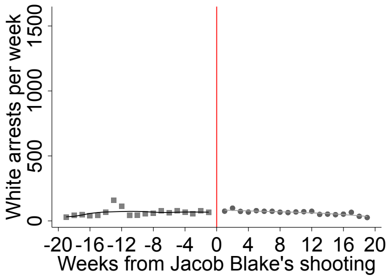

Table S1: Regression-adjusted differences in of trust in the police

	Dependent variable: Expressed great trust in the police (0,1)								
	(1)	(2)	(3)	(4)	(5)	(6)	(7)	(8)	(9)
Race/Ethnicity									
Black	-0.184***								-0.226***
	(0.023)								(0.025)
Asian	-0.119***								-0.081**
	(0.039)								(0.038)
Hispanic	-0.110***								-0.102***
	(0.028)								(0.030)
Gender									
Male		0.032							0.023
		(0.022)							(0.021)
Age									
18-29			-0.192***						-0.226***
			(0.031)						(0.038)
30-44			-0.160***						-0.185***
			(0.029)						(0.036)
45-64			-0.077**						-0.098***
			(0.031)						(0.033)
Education									
College degree or more				0.005					-0.037
				(0.021)					(0.025)
Criminal justice system contact									
Arrested, booked, or jailed					-0.100***				-0.076**
					(0.029)				(0.031)
Years in the neighborhood									
More than 5 years						0.094***			0.034
						(0.020)			(0.023)
Home ownership status									
Homeowner							0.089***		0.015
							(0.021)		(0.023)
Employment status									
Employed								-0.023	0.028
								(0.021)	(0.024)
Constant	0.300***	0.212***	0.326***	0.221***	0.233***	0.168***	0.185***	0.236***	0.406***
	(0.016)	(0.013)	(0.024)	(0.015)	(0.011)	(0.015)	(0.013)	(0.016)	(0.038)
Observations	1,616	1,616	1,616	1,616	1,616	1,616	1,616	1,616	1,616
Adj. R^2	0.036	0.001	0.030	0.000	0.005	0.012	0.011	0.001	0.086

Data are from the 2020 Healthy Chicago Survey.
The included sample includes all survey respondents interviewed before the shooting of Jacob Blake.
Standard errors are robust to heteroscedasticity. $* < 0.10$, $** < 0.05$, $*** < 0.01$

Table S2: Mean trust in the police and number of observations for different subgroups used in the analyses

	**4w-1w before**<br>mean outcome<br>(observations)	**1w-4w after**<br>mean outcome<br>(observations)
Black, All	0.50<br>(430)	0.45<br>(154)
White, All	0.69<br>(730)	0.67<br>(209)
Black, 18-44	0.31<br>(115)	0.21<br>(47)
White, 18-44	0.56<br>(383)	0.53<br>(106)
Black, 45 and older	0.58<br>(314)	0.55<br>(107)
White, 45 and older	0.84<br>(347)	0.83<br>(103)
Black, Police contact	0.37<br>(75)	0.23<br>(26)
White, Police contact	0.61<br>(49)	0.59<br>(27)
Black, No police contact	0.53<br>(355)	0.49<br>(128)
White, No police contact	0.69<br>(681)	0.69<br>(182)
Black, Male	0.47<br>(123)	0.40<br>(50)
White, Male	0.69<br>(299)	0.64<br>(89)
Black, Female	0.52<br>(294)	0.47<br>(101)
White, Female	0.70<br>(424)	0.70<br>(118)

Results for Hispanics

Figure S3: Changes in trust in law enforcement after Jacob Blake's shooting among Hispanics

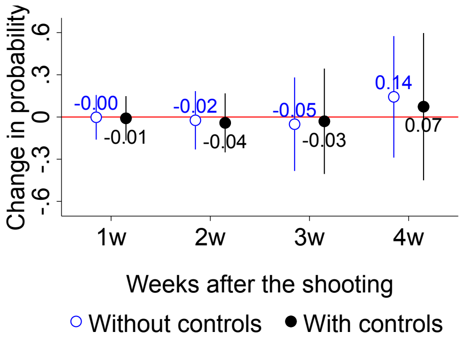

Data are from the 2020 HCS survey. The survey question used to create the measure of trust in law enforcement is *"To what extent do you trust your law enforcement agency?"* We code answers to this question as binary, taking on value 1 for those that responded *"a great extent"* or *"somewhat"* and 0 for the rest. Each pair of point estimates shows the change in the probability of expressing trust for those surveyed one, two, three, and four weeks after Jacob Blake's shooting, relative to the pool of survey respondents interviewed in the four weeks prior. Within each pair of estimates, we show results with and without the set of controls listed in Table 2. Confidence intervals are at the 95% level with standard errors robust to heteroskedasticity.

Additional results by age, gender, and educational attainment

Figure S4: Changes in trust in law enforcement after Jacob Blake's shooting

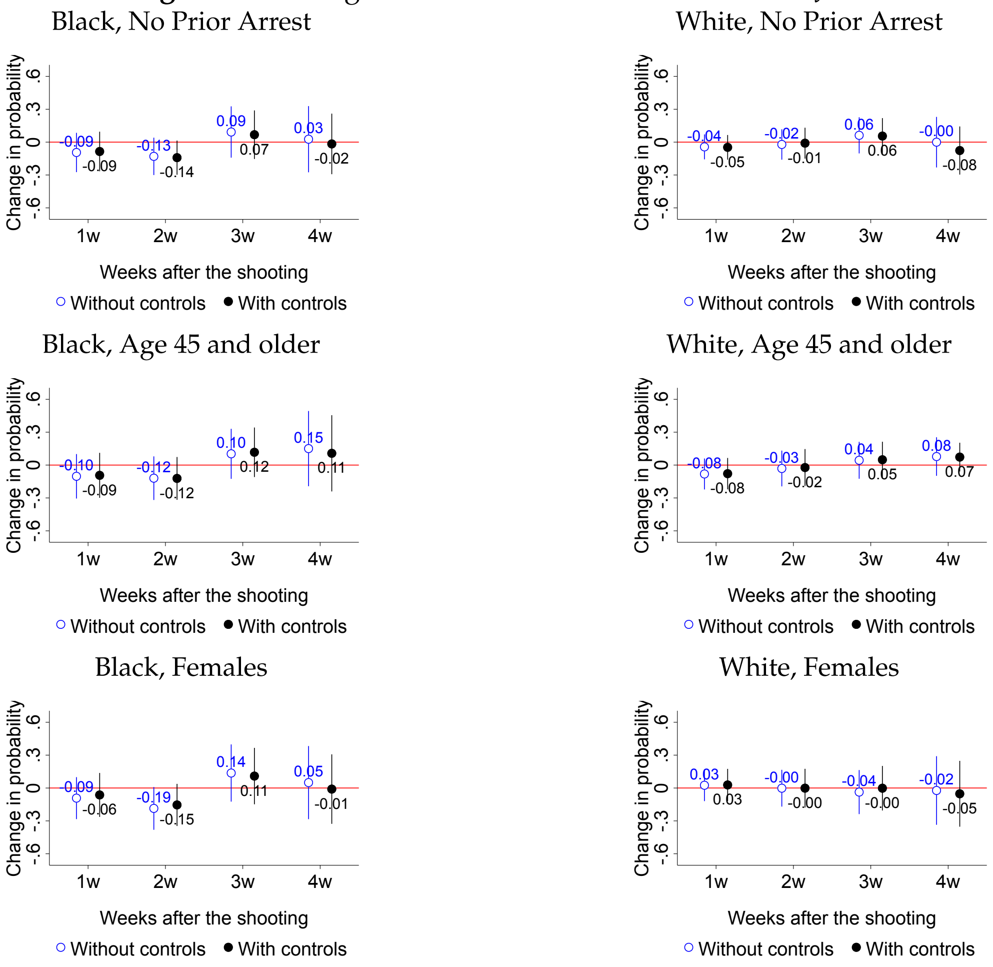

Data are from the 2020 HCS survey. The survey question used to create the measure of trust in law enforcement is *"To what extent do you trust your law enforcement agency?"* We code answers to this question as binary, taking on value 1 for those that responded *"a great extent"* or *"somewhat"* and 0 for the rest. Each pair of point estimates shows the change in the probability of expressing trust for those surveyed one, two, three, and four weeks after Jacob Blake's shooting, relative to the pool of survey respondents interviewed in the four weeks prior. Within each pair of estimates, we show results with and without the set of controls listed in Table 2. Confidence intervals are at the 95% level with standard errors robust to heteroskedasticity.

Effects in 2-day windows

Figure S5: Changes in trust in law enforcement after Jacob Blake's shooting modeled in 2-day windows

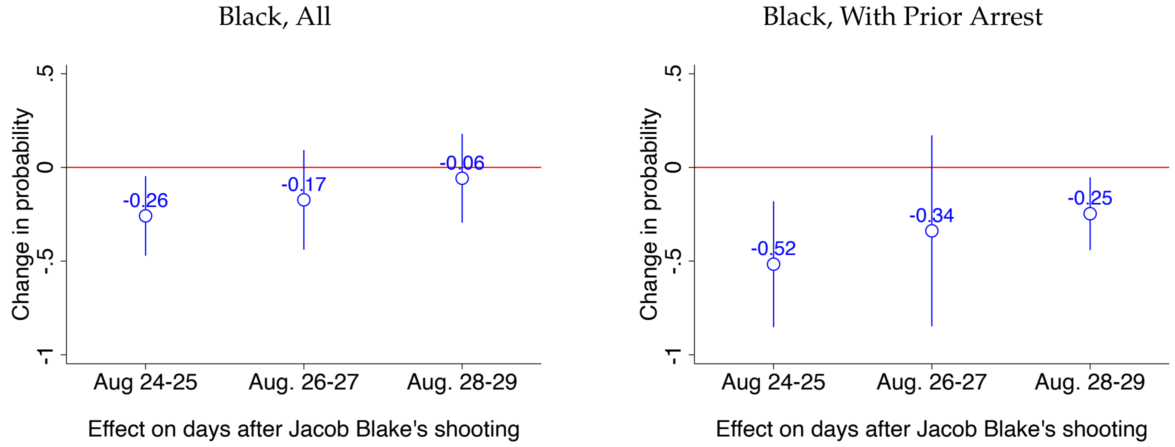

Data are from the 2020 HCS survey. The two survey questions used to create the measures of trust are *"To what extent do you trust your law enforcement agency?"* We code answers to these questions as binary, taking on value 1 for those that responded *"a great extent"* or *"somewhat"* and 0 for the rest. Each pair of point estimates shows the change in the probability of expressing trust for those surveyed after Jacob Blake's shooting in 2-day intervals, relative to the pool of survey respondents interviewed in the four weeks prior. Confidence intervals are at the 95% level with standard errors robust to heteroskedasticity.

Falsification Tests

Figure S6: Changes in the probability of drinking soda at least once per day

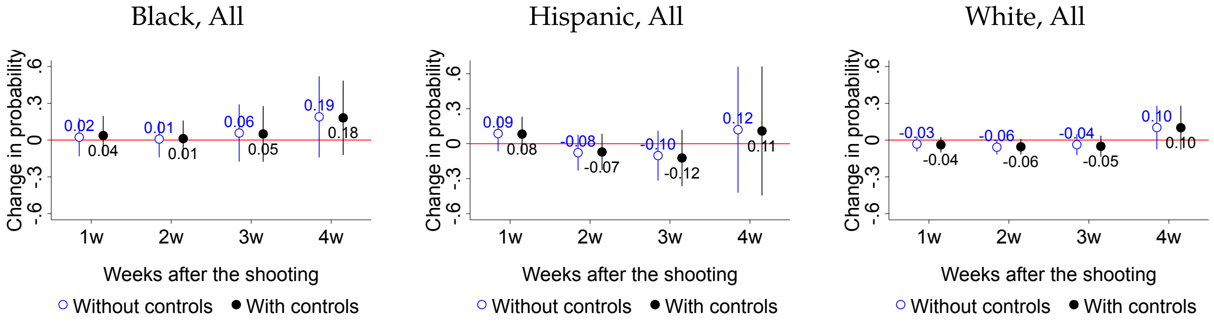

Data are from the 2020 HCS survey. We use answers to the question *"During the past 30 days, how many regular soda or pop or other sweetened drinks like sweetened iced tea, sports drinks, fruit punch or other fruit-flavored drinks have you had per day?"* to measure the probability of soda consumption. Each pair of point estimates shows the change in the probability of having had at least one soda drink per day for those surveyed one, two, three, and four weeks after Jacob Blake's shooting, relative to the pool of survey respondents interviewed in the four weeks prior. Within each pair of estimates, we show results with and without the set of controls listed in Table 2. Confidence intervals are at the 95% level with standard errors robust to heteroskedasticity.
